# Monitoring of Poyang lake water for sewage contamination using human enteric viruses as an indicator

**DOI:** 10.1186/s12985-017-0916-0

**Published:** 2018-01-05

**Authors:** Hui Zhu, Fang Yuan, Zhaokang Yuan, Rong Liu, Fei Xie, Ling Huang, Xiaojun Liu, Xiaoqing Jiang, Jian Wang, Qunying Xu, Zhiqiang Shen, Donghan Liu, Ronghao Zhang, Yuanan Lu

**Affiliations:** 1 School of Public Health, Jiangxi Province Key Laboratory of Preventive Medicine, Nanchang University, Jiangxi, 330006 People’s Republic of China; 2 Office of Public Health Studies, University of Hawaii at Mānoa, Honolulu, Hawai’i 96822 USA; 3 Tianjin Institute of Health and Environmental Medicine, Tianjin Key Laboratory of Risk Assessment and Control Technology for Environment and Food Safety, Tianjin, 300050 China

**Keywords:** Enteric virus, RT-PCR, Water quality indicator, Poyang Lake

## Abstract

**Background:**

Recreational water contaminated with fecal pollution poses a great public health concern, as fecal waste may cause serious waterborne illnesses. Current recreational water standards using fecal indicator bacteria (FIB) have their limitations for human protection especially in developing countries such as China.

**Methods:**

To explore the potential use of enteric viruses as a potential indicator of fecal contamination, four viruses: norovirus geno-groups I and II, enteroviruses, and adenoviruses were tested in this study using molecular detection methods and sensitive RT-PC developed in the University of Hawaii. Water samples were also tested for FIB in order to determine their association with enteric virus detection.

**Results:**

All sample sites tested positive for four enteric viruses. Human enterovirus (58%) and adenovirus (67%) were more frequently detected from these six sites, followed by norovirus I (50%) and norovirus II (38%). Six sampling sites all met the level-I water quality of GB3838–2002 criteria in microbiological level, but they all tested positive for enteric viruses.

**Conclusion:**

These findings indicate the current sewage contamination of Poyang Lake and also support the essential need of additional indicator such as human enteric viruses for enhanced monitoring of water quality since the presence of enteric viruses does not always correlate with fecal bacterial indicator detection.

## Background

Occurrence of waterborne illnesses has been one of major public health concerns in low-, middle-income, and high-income countries. Fecal contamination is the main cause of waterborne diseases, because human pathogens such as bacteria, viruses and protozoa are able to survive in the polluted water and infect recreational water users [1]. With the recently increased outbreaks of viral diseases reported from recreational water, it is necessary to establish more effective detection methods for enhanced monitoring of water quality [2, 3]. Today, most countries around world are mainly basing on bacterial indicators to monitor both recreational and drinking water, In the Unites States, Environmental Protection Agency (EPA) started to measure total coliform and fecal coliform as water quality indicators in 1976 and has now focused on measuring fecal indicator bacteria (FIB) such as *E. coli for freshwater* and enterococci for sea and salty recreational waters to assessing water contamination and risk [4]. In China, total bacterial count and total coliform have been chosen as bacterial character indicators for surface water monitoring. However, bacteria indicators cannot always be a reliable indicator system to monitor water quality since some studies have shown that some waters met the bacteria standards but low number of pathogenic virus was still detected, resulting in water-borne diseases [5–7]. Recent interest has been focusing on using human enteric viruses (HEV) as a potential indicator system for water quality monitoring and this indicator system has already been used as a regular indicator for water monitoring in the European Union countries since 1980’s [8, 9].

Human enteric viruses are recognized as common etiological agents for waterborne diseases outbreak and these human pathogens have been detected in recreational water in many countries around the world [6, 10–12]. Human enteric viruses represent a diverse group including members from different viral families *Picornaviridae* (polioviruses, enteroviruses, coxsakieviruses, hepatitis A viruses, echoviruses), *Calicividae* (noroviruses, caliciviruses, astroviruses), *Adenoviridae* (adenoviruses), and *Reoviridae* (rotaviruses) [13]. Human enteric viruses are transmitted through fecal-oral route and they usually cause waterborne diseases due to their resistant to heat and UV light inactivation in water and their low infection dose [14]. Enteric viruses usually cause diarrhea and gastroenteritis, but they may also cause respiratory infection, conjunctivitis and even diseases that have high mortality rate, such as aseptic meningitis and encephalitis [15]. It was reported that there are twenty-one gastroenteritis breakouts, and twenty of them due to noroviral infection between December 2016 and January 2017 in Beijing, China. In the United States, there were estimated 2536 persons infected among a total of 65 water-borne diseases outbreaks associated with recreational water reported from 23 states during 2001–2002, and five recreational water associated outbreaks of gastroenteritis were attributed by noroviruses, causing illness of 146 persons [16].

Compared to bacteria, HEV have several specific characteristics suitable to be considered for water quality monitoring: The first is that the dose of human enteric viruses for causing disease is much lower than that of the bacteria, in some cases, only one infectious viral unit could cause illness in human. A risk assessment conducted in 1993 showed that the illness risk was much less for bacteria than viruses when the same number of pathogens is exposed [14]. Secondly, HEV are more stable and persistent than bacteria in the environment waters and sewage. It is known that enteric viruses could survival longer in some extreme situations such as pH value ranging from 3 to 10 and low temperature [17]. Due to their small size, viruses may escape from some wastewater treatment processes such as sedimentation, activated carbon treatment and oxidation ponds [18]. It was also shown that the stable DNA or RNA structures of HEV could lead them to survive from UV treatment [14].

Therefore, HEV have shown a great promise to be considered as a potential indicator for monitoring water quality related to the fecal contamination. One of the major challenges of using HEV to monitor water quality is that viruses often occur at low levels in the water environment, and thus it requires highly sensitive concentration and optimized laboratory methods for effective detection of human pathogens in waters [19]. Recently, highly effective method have been established for enhanced concentration and detection of enteric viruses at the university of Hawaii, and these newly established protocols have been applied for successful detection of HEV from different environmental waters [19–21]. This study is aimed to utilize these newly established laboratory methods to conduct a survey check human sewage contamination of Poyang Lake, the largest fresh water lake in China which is surrounding Nanchang city. Results from this study will not only be valuable for the local government to understand for the first time present contamination of Poyang water from human sewage, but also will testified the methodologies developed in USA Hawaii are applicable to environmental waters in other regions. Parallel to this study, we carried out fecal indicator bacteria (FIB) specifically total coliform (TC) and fecal coliform (FC) analysis in order to better reveal whether there is the presence of detection correlation between enteric viruses and FIB.

## Methods

### Description of sites

**Fig. 1:**
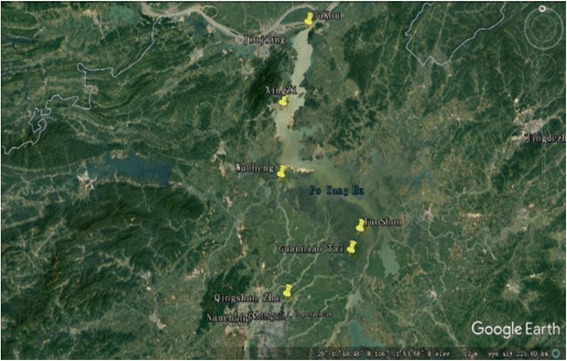
Map showing six sampling sites along Poyang Lake for this study. Water samples were collected monthly from each of these six locations of the lake between October 2016 and January 2017

Poyang Lake is the biggest fresh water lake in China and located in the north of Jiangxi Province, and to the middle and the lower reaches of the Yangtze River. This lake is connected with five main rivers including Gan River, Hu River, Xin River, Rao River, and Xiu River. Because its surroundings and natural resources, Poyang Lake provides local residents nearby opportunity for economic sources through fishing and the majority of drinking water. Therefore, the water quality of Poyang Lake is becoming a serious public health concern. In this study, water samples were taken from six locations along the Poyang Lake considering factors such as human sources and environmental impact (Fig. 1): the site Qingshanzha (1.8 million inhabitants nearby) located in Gan River which is selected since it is an important domestic wasterwater outlet (the rate of sewage treatment is 89%) from urban area in Nanchang; the site Guanniaotai and Tuoshan belong to Nanjishan Water of the southwest of Poyang Lake (total 3000 inhabitants), which are selected because of their kinds of biological resources especially migrant birds that may excrete feces into water; the site Xingzi (0.21 million inhabitants) and Wucheng (0.015 million inhabitants) are selected for their citizens live on Poyang Lake and visitors; and the site Dukou (0.29 million inhabitants), is the downstream of Poyang Lake, flows into Yangtze River, which may affected by upriver water quality. The site descriptions are summarized in Table 1. The water temperature of Poyang Lake was measured every sampling time for each of these six sites.

**Table 1:** Sites sampled in the Poyang Lake

Location	Details	GPS coordinates	
		Latitude Longitude	
Qing Shan Zha	In the south branch of Gan River, nearly the city of Nanchang	28°43′28”	115°55′47”
Guan Niao Tai	Belong to Jiangxi Provincial nature reserve, in the southwestern of Poyang Lake, Nanchang	28°41′13”	115°53′56”
Tuo Shan	Belong to Jiangxi Provincial nature reserve, in the southwestern of Poyang Lake, Nanchang	28°56′17”	116°21′12”
Wu Cheng	In the north-central of Poyang Lake, Jiujiang City	29°26′17”	116°2′41”
Xing Zi	In the north of Jiangxi Province, Jiujiang City	29°26′17”	116°2′42”
Du Kou	The only junction of Poyang Lake and Yangtze River in Jiujiang City	29°44′47”	116°12′56”

In this study, each site was monitored for four months from October of 2016 to January of 2017. Water samples were collected in the morning with sterilized 10-L polypropylene containers from the 10% hydrochloric acid (HCL) bath treatment as described previously [19, 20]. Samples were transported on ice to the laboratory of Nanchang University and processed for viral detection immediately. One Liter of water samples from each site was tested for HEV and another liter was for fecal indicator bacteria, respectively

### Sample concentration, nucleic acid extraction and RT-PCR

Sample concentration, nucleic acid isolation, and RT-PCR were performed using the protocols established previously in this laboratory that showed high efficiency of viral recovery from environmental water samples [19, 21–23]. In brief, 5 ml of 5 mol/L Magnesium Chloride was added to one liter of freshwater sample (final concentration of 25 mM) at the laboratory, and the sample was allowed to set for 5 min at room temperature before filtration. Samples were concentrated using negatively-charged type HA filter membranes (EMD Millipore, Billerica, MA, USA) with a 0.45 μm pore size and 47 mm in diameter under vacuum pump filtration, in a filtration-based method described previously [19]. One-liter water sample was filtered through for each fresh water sample, except when filters clog, filtration could take up to more than one hour, and the final amount of filtrated water was recorded. Nucleic acids were extracted from the recovered membranes using the Power Water® RNA Isolation Kit (MoBio Laboratories, Inc., Carlsbad, CA, USA), according to a modified protocol [20]. The final volume for each sample extraction was 60 μL, of which an aliquot 15 eluent was stored at −80 °C for viral DNA assays. The remaining 45 μL was used for RNA extraction by removing residual DNA through treating the extract with 5 μL of 10X DNase I buffer and 4 μL of DNase I. Following incubation at RT for 15–20 min. 0.75 μL of 0.5 M EDTA was added to the extract to protect RNA when DNase I was heated inactivate at 75 °C for 5 min. The recovered RNA was kept at −20 °C until use. RNA was subject to RT-PCR using the Easy Script® One-Step gDNA Removal and cDNA Synthesis Super Mix (Transgene, China). Reverse transcriptase PCR (RT-PCR) was used previous standardization and optimization as follows: 1 μL of Random Primer, 10 μL of 2 × ES Reaction Mix and 1 μL of Easy Script® RT/RI Enzyme Mix were added to 8 μL of sample RNA. Then cDNA samples were stored at 4 °C until use.

### PCR detection

PCR was conducted using the protocol described previously [19, 21] and tested for the following four enteric viruses: norovirus geno-groups I and II, enterovirus, and adenovirus. PCR conditions and primer set for each virus detection are summarized in Table 2. Both negative and positive controls were included for each PCR performance. In particular, deionized water and viral plasmid DNA prepared previously for each of the four viruses were used as negative and positive controls, respectively. The first PCR product was used as template for the second amplification under the same PCR condition. Results were visualized using gel electrophoresis on a 2% agarose gel stained with ethidium bromide.

**Table 2:** List of PCR primers used in this study with optimal amplification conditions

Virus	Primer name	Primer Sequence (5′ → 3′)	Length (bp)	Amplification condition				Reference
				TA	Mg (mM)	Primer (μM)	BSA	
NoV GI	QNIF4	CGCTGGATGCGNTTCCAT	8 6	58	2.0	0.4	Yes	Loisy et al., 2005
	NV1LCR	CCTTAGACGCCATCATCATTTAC						
NoV GII	COG2F	CARGARBCNATGTTYAGRTGGATGAG	9 7	58	2.0	0.8	Yes	Kageyama et al., 2003
	COG2R	TCGACGCCATCTTCATTCACA						
EoV	EQ-1	ACATGGTGTGAAGAGTCTATTGAGCT	1 4 2	60	1.5	0.6	Yes	Dierssen et al., 2008
	EQ-2	CCAAAGTAGTCGGTTCCGC						
AdV	ADV-F	GCCACGGTGGGGTTTCTAAACTT	1 3 2	54	1.5	0.6	Yes	Gunson et al., 2009
	ADV-R	GCCCCAGTGGTCTTACATGCACATC						

^a^NoV GI, human norovirus genotype I; NoV GII, human norovirus genotype II; EoV, human enterovirus; AdV, human adenovirus

### Sequencing

To verify accurate DNA amplification and viral detection, 51 PCR products from the nest PCR were cloned using TA Cloning kit purchased from Invitrogeen and sent to the Beijing Genomics Institute (BGI) for two directional sequencing. The sequencing data were jointed and compared to the available nucleic acid sequences in the National Center for Biotechnology Information (NCBI) database using Basic Local Alignment Search Tool (BLAST).

### Microbial detection and evaluation criteria

The bacteriological analyses were done according to the filter membrane method of GB/T 5750.12–2006 《Standard Examination Methods for Drinking Water》of China. The detected indicators including Aerobic bacterial Count (ACC), total coliform (TC), fecal coliform (FC) and *E. coli*. One milliliter of different dilution water were passed through 0.45 μM pore size, 39 mm diameter Millipore membrane filters, which were placed onto different culture media: ACC: Nutrient Agar, incubated at 36 °C for 48 h; TC: Endo Agar, incubated at 36 °C for 18–24 h; FC: MFC medium, incubated at 44.5 °C for 24 h; and *E. coli*: MUG medium, incubated at 44.5 °C for 24 h.

According to the following two standards to evaluating the water functions: (1). The average TC of source water in GJ3020–1993 《Standard Examination Methods for Drinking Water》 is 1000 CFU/L for I level water, 10,000 CFU/L for II level water. (2). GB3838–2002《Standards of surface water quality》for FC: I to IV level water quality limit is 200, 2000, 1 × 10^4^, 2 × 10^4^, 4 × 10^4^/L.

### Statistical analysis

Statistical analysis was carried out using SPSS version 19.0. The Spearman rank correlation was used to test the relationship between bacterial indicators and enteric viruses. *P*-value below 0.05 was considered statistically significant, and all *p*-values were given as two-tailed.

## Results

### RT-PCR detection

**Fig. 2:**
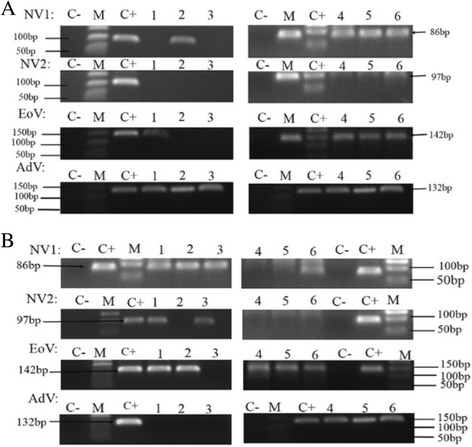
Agarose gel electrophoresis of RT-PCR and PCR detection of four enteric viruses from six sampling sites of Poyang Lake. The image of enteric virus detection in October (**a**) and November (**b**) in 2016. NoV GI, human norovirus genotype I; NoV GII, human norovirus genotype II; EoV, human enterovirus; AdV, human adenovirus. Lanes: 1 = Guan Niao Tai, 2 = Tuo Mountain, 3 = Qing Shan Zha, 4 = Du Kou, 5 = Xing Zi, and 6 = Wu City. Lanes M = 50 bp DNA marker; C+ = positive control; Lane C- = negative control containing no template

A total of twenty-four water samples were collected from six different sites along the Poyang Lake and analyzed. All sample sites tested positive for more than two enteric viruses during the 4-month study period. All these 4 HEV were tested positive from all the 6 sites at least once (Fig. 2). Nineteen of twenty-four samples (95%) tested positive for two or more viruses. As shown in Table 3, human enterovirus (58%) and adenovirus (67%) were more frequently detected from these six sites, followed by were norovirus I (50%) and norovirus II (38%). Sixteen of twenty-four water samples were tested positive for adenovirus, followed by enterovirus (14/24) and norovirus geno-group I (12/24). Under the described laboratory conditions, human norovirus geno-group II was tested positive in 9 of the 24 samples representing the least commonly detected virus in this study.

**Table 3:** Detection of human enteric viruses in water samples of Poyang Lake, Jiangxi, China

Sampling		NoV GI	NoV GII	EoV	AdV
Date	Site*				
October 10	1	–	–	–	+
	2	–	–	+	+
	3	+	–	–	+
October 28	4	+	–	+	+
	5	+	–	+	+
	6	+	–	+	+
November 10	1	+	+	–	–
	2	+	+	+	–
	3	+	–	+	–
November 28	4	+	–	+	*+*
	5	–	–	+	+
	6	–	–	+	+
December 10	1	+	+	–	–
	2	+	+	–	–
	3	+	–	–	+
December 28	4	–	+	–	+
	5	–	–	–	+
	6	–	+	+	+
January 10	1	–	–	+	–
	2	–	–	–	–
	3	–	–	–	–
January 28	4	+	+	+	+
	5	–	+	+	+
	6	–	+	+	+
Total		12	9	14	16

* 1 -Qing shan zha; 2 - Guan Niao Tai; 3 - Tuo Shan; 4 -Wu Cheng; 5 - Xing Zi; 6 - Du Kou

### Sequencing

All the selected PCR amplicons were sequenced viral detection verification and confirmed these positive detections were correct amplification as the viruses of interest. Several viral subtypes of enteric viruses were identified in Poyang Lake including Norovirus strains of GI.9, GI.7, GI.Toyama, GII.NEC, GII.Seoul, GII.CC, GII.Hu, and GII.CC, and human adenovirus type 41. Several other human enteric viruses were also detected in this study including human echovirus type 11, 25, E29 and human coxsackievirus B3. Full sequencing results were summarized in Table 4.

**Table 4:** Sequence results of amplified PCR fragments

Site	Blast	Matched DNA	E value	Identity	Sequence
3.2	KC911703.1	Norovirus Hu/GII/NEC-365/THA/	5.00E-10	98%	ATAATCATTACCCAAGTTTAAATGAAGAGGAGACGCATCTGGCTCCCAGTTTTGTGAAT GAAGATGGC
1.2	EU151450.1	Swine vesicular disease virus strain SVDV Itl. 1–92	5.00E-33	96%	CCATGGCTAGGACTGACTACTTTGATACGGCTAATCCTCACTCGCGTGAGCAGATACCCA CACACCAGTGGGCAGTCTGTCGTAATGGGCAACTCTGCAGCGGAACCGACTACTTTGG
1.3; 2.4	GU236215.1	Human echovirus 11 isolate 39,351.82 5’ UTR	6.00E-42	100%	AAGGTGAGGACCAGTACTCCTGAATGCGGCTAATCCTAACTGCGGAGCAGATACCCAC ACACCAGTGGGCAGTCTGTCGTAATGGGCAACTCTGCAGCGGAACCGACTACTTTGG
1.1	KX458103.1	Norovirus GI.9 strain	1.00E-09	98%	GGGGGTGGCGTCTAAGACATCTCCTACCCGATTATGTAAATGATGATGGCGTCTAAGGA
2.6	KF303071.1	Human adenovirus 41 isolate 10–4851	2.00E-31	98%	TGGGAACCTGCCTTAAGTCGATATCTCGTGGCGCGGGCAAACTGCACCAGGCCCGGA CTCAGATACTCCGAGGCGTCCTGCCCGGCGATGTGCATGTAAGACCACTGGGGCAG
2.5	GU236272.1	Human echovirus 25 isolate 06.048.1621 5’ UTR	1.00E-40	99%	GAGGACGAACACAGACCAACCGCCCACTGGTGTGTGGGTATCTGCTCCGCAGTTAGGAT TAGCCGCATTCAGGGGCCGGAGGATTACCAATTAGCTCAATAGACTCTTCACACCATGTAA
2.5; 4.6; 4.5; 4.4	LT628548.1	Human adenovirus 41 partial Hexon gene for Hexon gene pseudogene, strain Muonio/11V1867_3/2012/FIN	5.00E-32	97%	TAGGCCTTGGTCTTAATTCGATATCTGGTGGCGCGGGCAAACTGCACCAGGCCCGGACT CAGATACTCCGAGGCGTCCTGCCCGGCGATGTGCATGTAAGACCACTGGGGCAG
3.2; 5.3	JX412882.1	Human adenovirus 41 isolate CR6724 hexon gene	5.00E-37	100%	TGCTGGCGAGTCGTATCGGTGGCGCGGGCAAACTGCACCAGGCCCGGACTCAGATA CTCCGAGGCGTCCTGCCCGGCGATGTGCATGTAAGACCACTGGGGCACA
3.3; 4.4	AB112114.1	Human norovirus Saitama gene for RNA dependent RNA polymerase,	3.00E-15	100%	AGGGGCCGTGATTGCGATCTCCTGTCCACAAGCTCAAGTCATGGAACCGCATCCAGCGA
3.3	JX412882.1	Human adenovirus 41 isolate CR6724 hexon gene	5.00E-37	100%	TAGACGGCCGAGTCGTATCGGTGGCGCGGGCAAACTGCACCAGGCCCGGACTCAGATA CTCCGAGGCGTCCTGCCCGGCGATGTGCATGTAAGACCACTGGGGCACA
4.6	KX907729.1	Norovirus Hu/USA/2011/GI.P7_GI.7/CS5567	5.00E-13	98%	GGTAGGCGTCTGATCGCACTCTCCTACCCGATTATGTAAATGATGATGGCGTCTAAGGA
4.5	AB504701.1	Norovirus sewage/GI.7/Toyama/SW0703–10/2007/JP genes	1.00E-15	100%	ACGACTGTGATTGCGATCTCCTGTCCACAAGCTCAAGTCATGGAAACGCATCCAGCGA
3.1	JX439815.1	Norovirus Hu/GII/Seoul1055/KOR/2010	3.00E-12	98%	TGGTCCGACTCCGGCGCCGACAATCGGGCGCTCCGCAATCTGGCTCCCAGTTTTGTGA ATGAAGATGGCGTCGACT
4.5	KJ489414.1	Human coxsackievirus B3 strain 2679	1.00E-40	96%	GGTCATTCGACGACTGCGCACTGGTGTGTGGGTATCTGCTCCGCAGTTAGGATTAGCCG CATTCAGGGGCCGGAGGATTACCAATTAGCTCAATAGACTCTTCACACCATGTAGA
5.1; 5.2	LC147086.1	Norovirus Hu/GI/Toyama/outbreakApr3741 /2010/JP	9.00E-10	100%	GGCATGCGTCTGACGCATCTCCTACCCGATTATGTAAATGATGATGGCGTCTAAGAGA
5.1	KC911664.1	Norovirus Hu/GII/CC-685/THA/2006 RNA polymerase and capsid genes	2.00E-14	98%	TTGCCCCAGACGGGCATCGGTAGGTGGGGCGATCGCATCTGGCTCCCAGTTTTGTGA ATGAAGATGGCGTCGA
6.4	KX446506.1	Norovirus GII strain GII/Hu/NL/2010/GII	1.00E-21	97%	CAACGGATCTGAGCCGTGGGAGGGCGATCGCATCTGGCTCCCAGTTTTGTGAATGAAGATGGCGTCGA
6.6; 7.1; 8.6	JX976770.1	Human coxsackievirus B3 isolate CVB3SD2012CHN	1.00E-39	99%	ACCTTGCCGTACACAGTACACTGTATGCGGCTAATCCTAACTGCGGAGCAGATACC CACACACCAGTGGGCAGTCTGTCGTAATGGGCAACTCTGCAGCGGAACCGACTACTTTGGAC

### Detection of fecal indicator bacteria

The results of indicator bacteria are expressed as colony forming units (CFU) per 1 ML (Tables 5 and 6). The results show that TC varied between 0 and 2.2 × 10^3^ CFU/mL in November, and FC levels were between 0 and 2.4 × 10^5^ CFU/mL in November. Twenty-three of the 24 water samples tested met the TC I level of CJ3020–1993 water quality criteria. And only 1 water sample tested for FC did not meet I level water quality of GB3838–2002 criterion. Six sites were all met I level water quality of GB3838–2002 criterion in microbiological level, but these sites all tested positive for enteric viruses. The detection of majority of enteric viruses did not show significant correlation with the detection of bacteria indicators (Table 6).

**Table 5:** Detection of fecal indicator bacteria for the water samples of Poyang Lake

Sampling Site*	Aerobic bacterial Count (CFU/ml)				Total Coliform (CFU/100 ml)				Fecal Coliform (CFU/100 ml)			
	Oct.	Nov.	Dec.	Jan.	Oct.	Nov.	Dec.	Jan.	Oct..	Nov.	Dec.	Jan.
1	3.1 × 10^4^	1.4 × 10^4^	5.0 × 10^1^	5.0 × 10^1^	0	2.2 × 10^5^	0	0	0	2.4 × 10^5^	0	0
2	1.9 × 10^2^	1.2 × 10^2^	1.0 × 10^1^	0.5 × 10^1^	0	1 × 10^2^	0	0	0	1 × 10^2^	0	0
3	1.0 × 10^2^	9.5 × 10^2^	2.0 × 10^1^	2.0 × 10^1^	0.5 × 10^2^	0	0	0	0	0	0.5 × 10^3^	0.5 × 10^1^
4	5.1 × 10^4^	4.4 × 10^4^	3.7 × 10^4^	2.5 × 10^4^	1 × 10^2^	1 × 10^2^	0.5 × 10^2^	0	1 × 10^2^	1 × 10^2^	0	0
5	3.9 × 10^2^	3.0 × 10^2^	3.5 × 10^2^	2.8 × 10^2^	0.5 × 10^2^	0.5 × 10^2^	2.0 × 10^2^	0	1 × 10^2^	1 × 10^2^	0.5 × 10^2^	0
6	2.2 × 10^3^	1.6 × 10^3^	3.1 × 10^3^	3.9 × 10^2^	0	0	0	0	0.5 × 10^2^	0.5 × 10^2^	0	0

*1 -Qing Shan zha; 2 - Guan Niao Tai; 3 - Tuo Shan; 4 -Wu Cheng; 5 - Xing Zi; 6 - Du Kou

**Table 6:** Correlation of enteric viruses between bacteriological index in Poyang Lake (*p* < 0.05)

	Spearman’s correlation coefficients (*p* value is two-tailed)			
	NoV I	NoV II	EoV	AdV
TC	−0.503(0.196)	−0.353(0.260)	0.359(0.452)	0.052(0.921)
FC	0.078(0.878)	−0.685(0.016)	−0.462(0.113)	0.221(0.618)
*E. coli*	−0.334(0.326)	−0.587(0.088)	0.213(0.727)	−0.213(0.727)

## Discussion

Poyang Lake is known to be the source for fish, crayfish and other aquatic food products to local residents year around. However current pollution of the lake with various contaminants has raised a concern to the public. No test has been conducted to the lake water for sewage contamination and human pathogens such as enteric viruses. In this study, we tested six sampling sites along the lake for enteric virus contamination for the first time. We have shown that all these sample sites are positive for the enteric viruses, indicating a serious fecal contamination of Poyang Lake water. In particular, adenovirus was detected to be the most common one among the four HEV in this study. This finding is different from previous report from Hawaiian recreational waters [20, 21]. Adenoviruses are a DNA-containing enteric virus and they are important human pathogens [9], Incidences of human adenoviral infection are reported in Jiangxi and other regions of China. Frequent detection of adenoviruses in Poyang Lake indicates this pathogen remains to be a health threat to the local residents and the need of the local government to take effective measurement to prevent and control of adenoviral infection.

This study revealed that all the selected viruses were detected at least once from all the sampling sites and this might be an indication of substantial fecal pollution of the Poyang Lake. The pollutants to Poyang Lake water are generally attributed from two sources: firstly, the pollutant from five major river systems which are directly connected to Poyang Lake; and secondly, the pollutant entered into Poyang Lake from various sources including urban sewage, industrial wastewater, and agriculture [24]. Enteric viruses shed from human and animal faces and viral pathogens may be absorbed (bioaccumulation) and then excreted by shellfish into water when the water is contaminated with feces [25]. Among the selected sampling sites, Guan Niao Tai site is located in the southern district of Poyang Lake, belongs to Nanji Mountain Nuture Reserve, where there have abundant migratory birds in winter and a variety of shellfishes and hydrophytes in water [26, 27]. This site owns the same geographical feature as Wu Cheng and Xing Zi. Lots of visitors come to the area for recreation, which may explain the presence of enteric viruses in this site. Site Qing Shan Zha was found to be positive for all four enteric viruses in November, indicating the sewage pollution in this area is relatively more serious. Qing Shan Zha is the different from the other sites and it is more close to Nanchang urban area, indicating this site is more subjective to sewage contamination. In addition, this site is an important draining mouth in the past, and now becomes a main source of pollution in the south branch of Gan River that connects to Poyang Lake. Various garbage and white foam were observed from domestic sewage and industrial wastewater during all water sampling times, these contaminations may directly contribute to the positive detection of different types of human enteric viruses from this site [28].

The outbreak of waterborne disease caused by enteric viruses has frequently been reported worldwide, including China [29–31]. Positive detection of different enteric viruses in this study indicates the presence of sewage contamination to Poyang Lake, and potential water-borne disease to human. Although enteric viruses have recently been detected in freshwater lakes in Wuhan of China, this study represents the first report of detecting enteric viruses in Poyang Lake region, Jiangxi Province. It is important to realize that enteric viruses are a highly pathogenic pathogen and thus sewage contamination could lead to viral infection and serious illness to local residents [31]. Positive detection of enteric viruses in Poyang Lake has raised warning alert to local government and the urgent need to pay more attention to local environmental waters, particularly those used for recreation and aquatic food production. In particular, present findings have provided strong evidence to local government for taking immediate measurement to identify the source of present sewage contamination and thus effective approach could be developed to stop and prevent the sewage pollution to Poyang Lake in future.

Sequence analysis revealed the detection of several strains of these four viruses in this study, including our detection of Human adenovirus 41 which was associated with gastroenteritis in children [32], human echovirus 11 - a same strain detected in Hawaiian recreational water, and human echovirus 25, and three different strains of human norovirus geno-group II (Norovirus GI.7, GI.9, GII). Our results showed that some viral strains detected in Poyang Lake are the same as detected in Hawaii, but some others are different from the strains detected in Hawaii and Wuhan China using these same detection methods. In Pisa, Carducci A et al. found not only human adenovirus type 41 which the same viral strain detected in our study, but also found human adenovirus type 31, human adenovirus type 2 AJ293902, one strain of norovirus genotype I and three strains of genotype 2, which were different from our detected strains [33]. In Hebei China, NoV GII.17 had been found in water and had cause a gastroenteritis outbreak [34]. These findings indicate that the methods used in this study are not limited to detecting the target HEV strains present in Hawaii only, and application of these methods will also be capable of detecting other related enteric strains of the target viruses [1, 20, 21].

In this study, all individual water samples collected from the six sites were also tested for the presence of fecal indicator bacteria as comparison. The results of this test are along with enteric virus detection, confirming the presence of human sewage contamination of Poyang Lake water. It is interesting to note that some water samples met the safety level basing on bacteria standards but they were tested positive for enteric viruses. This indicates the disagreement for the water quality between the two indicator systems, indicating that the limitation of using TC and FC to monitor biological safety of recreational water. Also it was reported before that bacteria indicators may regrow in the tropical environment after being excreted from their host and most importantly, they are not always correctly relating with the presence of enteric viruses, which could pose a great threat to the public. Given the limitation of the current bacterial indicator, establishment of more sensitive viral concentration and detection methods and more validation test from different waters are necessary in order to facilitate the development of using enteric viruses as an alternative or complementary indicator along with fecal indicator bacteria (FIB) for enhanced monitoring of environmental water quality [13].

## Conclusions

Poyang Lake was assessed for human enteric virus for the first time and four different types of enteric viruses were tested using the methods recently established from University of Hawaii. Our detection result showed all the six sampling sites were positive for enteric viruses, which strongly suggests the presence of human sewage contamination of Poyang Lake water. Since the lake is currently used for fishing, drinking, swimming, boating and other recreational activities, disease prevention program needs to be established to protect the public. The present detection of enteric viruses was conducted totally basing on RT-PCR and PCR methods and thus the positive viral detection might not necessarily mean the presence of infectious viruses in the lake water, so does not inevitably mean that there is a risk to human health. However, positive detection of enteric virus is a clear indication of the sewage contamination of the lake, which indicates it is important and essential for local government to start to monitor the lake water for sewage contamination routinely in future, and also to search and identify the source of the present contamination. Thus future pollution of the lake water can be effectively stopped and eliminated. Current detection of enteric viruses together with bacterial detection in Poyang Lake also substantiates the current concept and necessity of using enteric viruses as an alternative and complementary indicator for enhanced monitoring of water quality and safety.

## Abbreviations

ACC: Aerobic bacterial count
Adv: Human adenovirus
BLAST: Basic local alignment search tool
CFU: Colony forming units
Eov: Human enterovirus
EPA: Environmental protection agency
FC: Fecal coliform
FIB: Fecal indicator bacteria
HCl: Hydrochloric acid
HEV: Human enteric virus
NoV GI: Human norovirus genotype I
NoV GII: Human norovirus genotype II
PCR: Polymerase chain reaction
RT-PCR: Reverse transcript-polymerase chain reaction
TC: Total coliform
